# Macrophage-associated prognostic modeling uncovers immunotherapy response mechanisms and defines HAGHL as a novel oncogenic driver in breast cancer

**DOI:** 10.3389/fimmu.2026.1776875

**Published:** 2026-03-26

**Authors:** Shengbin Pei, Wenlong Chen, Zheng Qu, Zheng Li, Xudong Zhang, Luxiao Zhang, Yazhe Yang, Yi Fang, You Meng

**Affiliations:** 1 Department of Breast Surgical Oncology, National Cancer Center/National Clinical Research Center for Cancer/Cancer Hospital, Chinese Academy of Medical Sciences and Peking Union Medical College, Beijing, China; 2 Department of Thyroid and Breast Surgery, Nanjing Medical University Affiliated Suzhou Hospital: Suzhou Municipal Hospital, Gusu School, Nanjing Medical University, Suzhou, China; 3 Department of Breast and Thyroid Surgery, Shanghai Tenth People’s Hospital, School of Medicine, Tongji University, Shanghai, China; 4 Department of General Surgery, People’s Hospital of Pingshun County, Changzhi, Shanxi, China

**Keywords:** breast cancer, HAGHL, immunotherapy, macrophages, prognostic

## Abstract

**Background:**

Macrophage−related genes (MRGs), a group of pivotal regulators governing macrophage differentiation, polarization, and function, have increasingly been recognized as critical modulators in tumor progression and immune evasion. However, the molecular expression profiles of MRGs and their intricate relationships with the immune microenvironment in breast cancer (BRCA) remain insufficiently investigated.

**Methods:**

We first used bulk RNA sequencing and single-cell RNA sequencing data from the TCGA and GEO databases, we analyzed the molecular expression patterns and clinical relevance of MRGs in BRCA. A prognostic model was developed using these genes, and the variations in the immune microenvironment between the high-risk and low-risk groups were evaluated. Additionally, the model’s predictive ability for immunotherapy response was assessed. Finally, we conducted *in vivo* and *in vitro* experiments to study the biological functions of HAGHL.

**Results:**

Multi-omics analysis identified a group of MRGs with prognostic value, leading to the successful development of a model that stratified BRCA patients into high- and low-risk categories. The model demonstrated high accuracy in predicting patient survival. Immune microenvironment-related analysis revealed significant differences between risk groups, and the model effectively predicted responses to immunotherapy. CellChat analysis suggested potential macrophage pathways in BRCA. Our results also show that HAGHL, as a carcinogenic factor in BRCA, knockdown can inhibit the proliferation and invasion of BRCA cells.

**Conclusions:**

We developed a prognostic model based on MRGs, which holds promise for predicting outcomes in BRCA patients and responses to immunotherapy. Our findings offer new insights and potential guidance for personalized treatment strategies in BRCA. Additionally, we identified HAGHL as a potential oncogene, laying the groundwork for future research.

## 1 Introduction

Breast cancer (BRCA) is among the most common cancers affecting women worldwide and is characterized by considerable variability (1–4). While conventional treatments such as surgery, chemotherapy, and radiotherapy play crucial roles in treating BRCA, the prognosis for advanced stages continues to be unfavorable (5). Despite the progress made through subtype-based therapies, substantial challenges and limitations remain in treatment outcomes (6). This highlights the urgent need for a more refined understanding of BRCA subtypes and the identification of specific therapeutic targets to enhance clinical decision-making. Immunotherapy has recently emerged as a promising approach, offering new hope for patients with BRCA mutations (7). It harnesses the body’s immune system to identify and eliminate tumor cells, primarily via immune checkpoint blockers, such as PD-1/PD-L1 inhibitors, and cancer vaccines (8, 9). In particular, immunotherapy has shown notable success in some cases of triple-negative breast cancer, especially when combined with chemotherapy (10). However, compared with other cancers, BRCA generally has a lower response rate to immunotherapy (11, 12). This disparity highlights the need for more comprehensive investigations into the molecular mechanisms and biomarkers that influence immunotherapy response, which are crucial for refining treatment strategies and enhancing patient outcomes (13).

Tumor-associated macrophages (TAMs) are an important component of the tumor microenvironment (TME) (14–16). By creating an immunosuppressive microenvironment, they play a crucial role in tumor progression and drug resistance (17, 18). Macrophages, as key members of both innate and adaptive immunity, not only participate in the body’s response to pathogens but also play an important role in maintaining the homeostasis of the internal environment (19, 20). MRGs are pivotal in macrophage differentiation, polarization, and function, and have gained prominence in tumor immunity and inflammatory disease research (21, 22). As key innate immune cells, macrophages adopt pro−inflammatory M1 or immunosuppressive M2 phenotypes under the control of these genes, shaping the TME and disease trajectory (23). In pathological states such as cancer, metabolic reprogramming and altered microenvironmental signals drive shifts in gene expression, regulating macrophage recruitment, activation, polarization, and the secretion of cytokines, chemokines, and metabolites that establish immunosuppressive or pro−inflammatory networks, thereby influencing tumor growth, invasion, and immune evasion (24, 25).

In BRCA, MRGs modulate tumor−associated immune cells; they can skew TAMs toward an M2 phenotype that fosters tumor growth, inhibits T−cell function, and weakens antitumor immunity (26, 27). Moreover, they suppress antigen−presenting cell and effector T−cell activity, altering sensitivity to immune checkpoint inhibitors and facilitating immune escape (28, 29). These multifaceted roles and TME interactions underscore the need for deeper investigation into macrophage−associated genes in BRCA (30).

From a clinical perspective, further stratification of BRCA patients is necessary to guide individualized and precise treatment (31). In this study, through integrative multiomics analysis, we subsequently investigated the molecular characteristics and clinical relevance of MRGs in BRCA. We developed a prognostic model based on MRGs and further investigated the complex crosstalk between these genes and the immune microenvironment in BRCA. Our model demonstrated strong predictive performance in estimating patient prognosis and immunotherapy response, offering valuable insights for the personalized treatment and management of BRCA patients in clinical practice. In addition, within the framework of the model genes, hydroxyacyl glutathione hydrolase (HAGHL) was characterized as an essential molecular component. We revealed that HAGHL emerges as a previously undescribed oncogenic factor related to immune evasion mechanisms.

## 2 Methods

### 2.1 Data collection

This research utilized a training cohort comprising RNA expression data from 1,098 BRCA patients, along with related clinical information sourced from the TCGA database (32). Multiple datasets were retrieved from the GEO database, including five bulk RNA-seq datasets (GSE20685 with 327 samples, GSE20711 with 92 samples, GSE42568 with 104 samples, GSE88770 with 117 samples, and GSE162228 with 109 samples) and one immunotherapy-related dataset (GSE103668 features 21 samples of TNBC patients who received treatment with cisplatin and bevacizumab in a neoadjuvant clinical trial) (33). Single-cell RNA sequencing data for BRCA were sourced from the GSE176078 dataset (34). This dataset contains single−cell RNA sequencing (scRNA−Seq) data from 26 untreated primary breast tumors, including 11 ER+, 5 HER2+, and 10 TNBC cases. Also included is the IMvigor210 cohort, a continuing international single−arm phase II trial assessing 5−year follow−up results of atezolizumab administered as first−line therapy in cisplatin−ineligible patients with metastatic urothelial carcinoma. We obtained a gene expression matrix and clinically informative data related to immunotherapy response from this cohort. In addition, a total of 585 MRGs were selected on the basis of the Gene Ontology database.

### 2.2 Consensus clustering analysis

We performed clustering analysis on the training set via the “ConsensusClusterPlus” package. Specifically, we utilized agglomerative PAM clustering on the basis of 1-Pearson correlation distances, resampling 80% of the samples over 10 iterations. The ideal number of clusters was assessed via an empirical cumulative distribution function plot and by evaluating the average consistency among the cluster groups (k value).

### 2.3 Differentially expressed gene analysis

The R package “Limma”, which is specifically designed for analyzing differentially expressed genes (DEGs) across distinct comparison groups, was used to screen for genes that showed significant differences in the training cohort. To identify DEGs between tumor and normal tissues in the training set, we set a condition of p < 0.05 and |log2FC| > 0.5. To narrow the range of our target genes, we set a condition of p < 0.05 and |log2FC| > 2 to screen for DEGs after clustering analysis was conducted. The “ggplot2” package was used to develop a volcano map for visualizing the distribution of DEGs.

### 2.4 Functional enrichment analysis

To explore specific biological signaling pathways among different clusters, we performed a thorough analysis via the “clusterprofiler” and “GSVA” software packages. For this study, we utilized gene sets from MsigDB version 7.0 and applied the GSVA algorithm to conduct an extensive evaluation of each gene set, investigating possible alterations in biological function across samples. Additionally, we employed GSEA to detect enriched pathways or gene sets on the basis of differential expression outcomes. The background gene set for this analysis was derived from the annotated gene sets of MsigDB, which served as a reference for the cluster pathways.

### 2.5 Construction and validation of the MRG-related risk signature

We performed consensus clustering using the “ConsensusClusterPlus” R package (v1.62.0) with the partitioning around medoids (PAM) algorithm and Euclidean distance. The number of clusters (k = 2–6) was evaluated based on the cumulative distribution function (CDF) and delta area plot, and k = 2 was selected as the optimal cluster number. DEGs between clusters were identified using the “limma” package with adjusted p < 0.05 as the threshold. To construct a prognostic signature, univariate Cox regression analysis was first performed to identify survival-associated genes (p < 0.05). These candidate genes were further subjected to LASSO Cox regression analysis using the “glmnet” R package (v4.1-8). Ten-fold cross-validation was conducted to determine the optimal penalty parameter (λ), and the λ value corresponding to the minimum mean cross-validated error (λmin) was selected. A random seed was applied to ensure reproducibility. Subsequently, multivariate Cox regression analysis was performed to obtain regression coefficients, and the risk score for each patient was calculated as follows: Risk score = Σ (Coef_i × Expression_i). Patients were divided into high- and low-risk groups according to the median risk score in the training cohort. The predictive performance of the model was evaluated using time-dependent ROC curve analysis via the “timeROC” package, and the AUC was calculated for 1-, 3-, and 5-year survival. K–M survival analysis with log-rank test was used to compare survival differences between groups. Univariate and multivariate Cox regression analyses were performed to determine whether the risk score was an independent prognostic factor after adjusting for clinical variables. A nomogram integrating risk score and clinical parameters (age, sex, and tumor stage) was constructed using the “rms” package, and its predictive accuracy was assessed using calibration curves based on 1,000 bootstrap resamples. External validation was conducted using independent GEO datasets and the IMvigor210 cohort to evaluate the robustness of the prognostic signature and its predictive value for immunotherapy response.

### 2.6 Immune cell infiltration analysis

In this study, we employed several algorithms, including CIBERSORT, MCP counter, and ESTIMATE, to assess immune cell infiltration levels within the TME, which were visualized via the “ComplexHeatmap” package (35).

### 2.7 Single−cell RNA−seq analysis

Breast cancer single-cell RNA-seq data were obtained from the GEO database under accession number GSE176078. Raw count matrices were imported into Seurat (v5.0.3) for downstream analysis. Cells were filtered using the following quality control criteria: cells with fewer than 200 detected genes or more than 6,000 genes were excluded, and cells with >10% mitochondrial gene expression were removed to eliminate low-quality or stressed cells (36). Genes expressed in fewer than three cells were also discarded. Data were normalized using the LogNormalize method with a scale factor of 10,000. Highly variable genes were identified using the FindVariableFeatures function (selection.method = vst, nfeatures = 2,000). Data were scaled using ScaleData, and PCA was performed based on highly variable genes. The top 30 principal components were selected for downstream analysis according to the elbow plot and JackStraw results. Batch effects were corrected using the IntegrateData function when multiple samples were present. Cells were clustered using the FindNeighbors and FindClusters functions (resolution = 0.5), and dimensional reduction was visualized using UMAP (37). Cell type annotation was performed based on canonical marker genes and previously published annotations. To analyze intercellular communication among integrated cell populations, CellChat (v1.6.1) was applied with default parameters to infer potential ligand-receptor interactions using a curated signaling database. We focused particularly on communication networks between epithelial cell subtypes and myeloid cells.

### 2.8 Cell culture and functional experiments

MDA-MB-231 and HCC1806 BRCA cell lines were sourced from the Shanghai Institute of Biochemistry and Cell Biology, CAS. Cells were maintained in DMEM or RPMI-1640 medium with 10% FBS. HAGHL and GAPDH primers were supplied by Qingdao Biotechnology Co., Ltd., and HAGHL−targeting shRNA lentiviruses were designed and produced by Genomeditech (Shanghai); sequences are listed in **Supplementary Table 1**. The colony formation, chamber, and plate cloning assay methods followed protocols from prior studies (38).

### 2.9 Statistical analysis​

Data processing, statistical analysis, and visualization were carried out using R (v4.2.0). All experiments were repeated three times independently, and results are shown as mean ± SD. Statistical tests were performed with GraphPad Prism 8.0 (La Jolla, CA, USA).

## 3 Results

### 3.1 Molecular characteristic analysis of MRGs in BRCA

**Figure 1:**
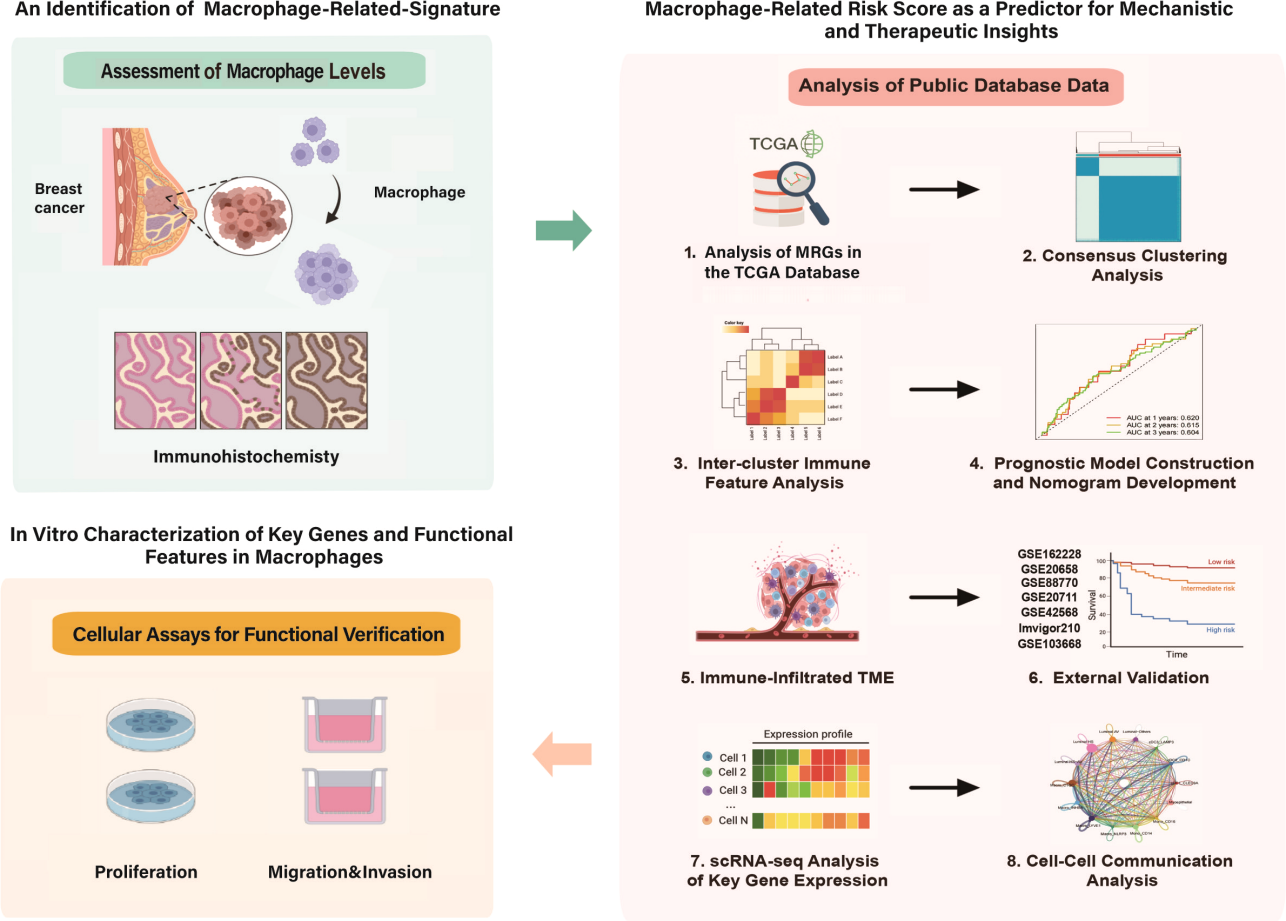
Workflow summary. This figure illustrates the workflow of the study, investigating the roles of MRGs and immune microenvironment remodeling in BRCA. The study employed multi-omics analysis to construct a macrophage-driven prognostic model for predicting clinical outcomes and immunotherapy response in BRCA patients. HAGHL was identified as a key oncogenic regulator, and its biological functions were systematically validated through comprehensive *in vitro* experiments, demonstrating its potential as both a prognostic biomarker and therapeutic target.

**Figure 2:**
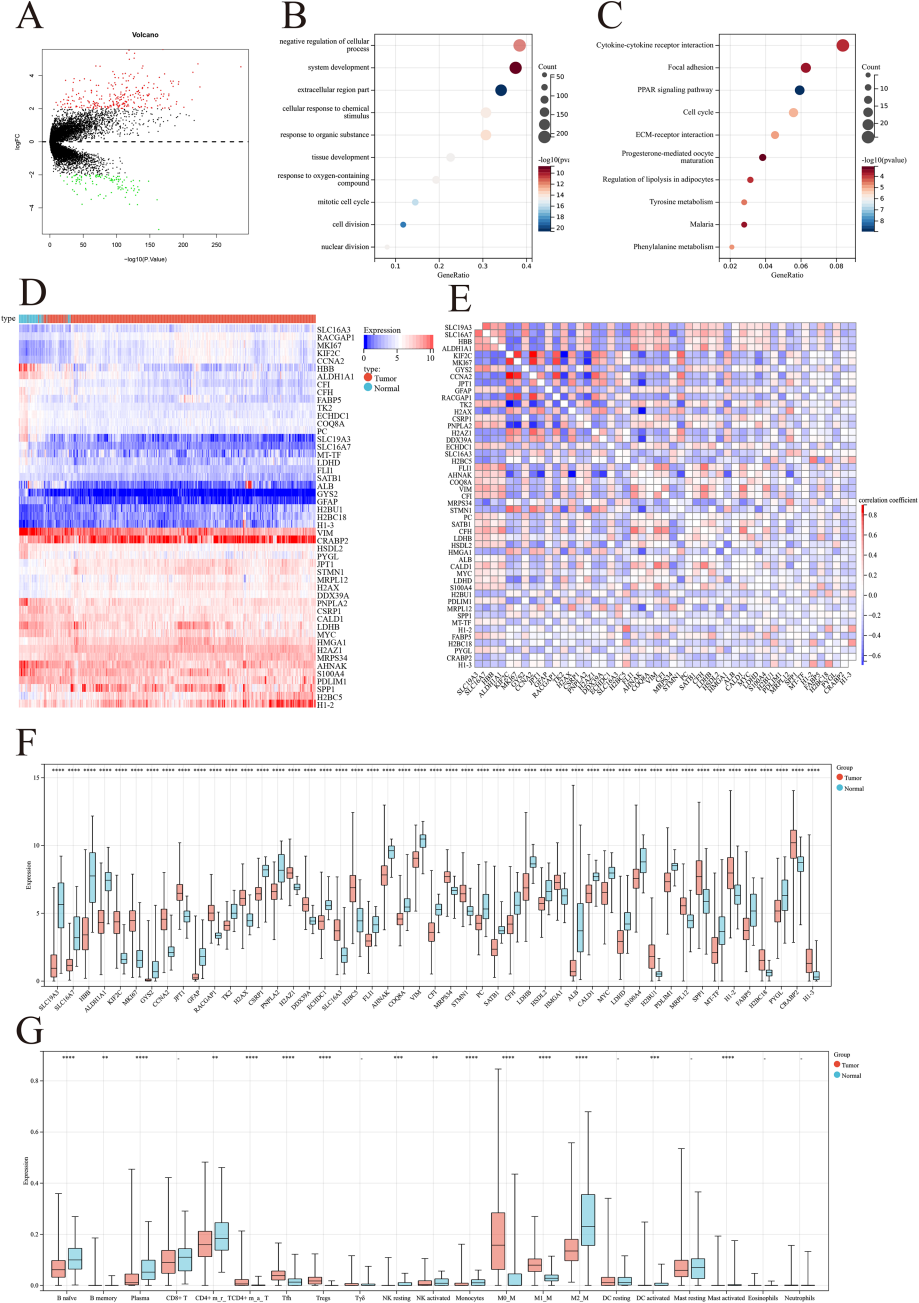
Molecular characteristics of lactate levels and MRGs in BRCA. **(A-C)** Differential gene expression analysis identified 612 macrophage-related genes (436 upregulated and 176 downregulated) in TCGA-BRCA cohort, mainly enriched in cell cycle, phylogenetic, and cytokine communication pathways. **(D-F)** Correlation analysis revealed distinct expression patterns of Macrophage-related genes: MKI67 positively correlated with KIF2C, CCNA2, and RACGAP1, but negatively correlated with TK2 and PNPLA2. **(G)** CIBERSORT analysis showed significant differences in immune cell infiltration between BRCA and control samples, particularly in M0 macrophages, M1 macrophages, T follicular helper cells, and regulatory T cells. **p<0.01; ***p<0.001; ****p<0.0001.

In recent years, Macrophage has been demonstrated to be widely present in various tumors and is involved in tumor development. We aimed to explore the significance of Macrophage in BRCA. **Figure 1** presents the overall framework of this study. Using large-scale transcriptomic data from the TCGA-BRCA cohort, we identified 612 differentially expressed genes, of which 436 genes were upregulated in tumors and 176 genes were downregulated (**Figure 2A**). These genes were enriched mainly in the cell cycle, phylogenetic, and cytokine communication pathways (**Figures 2B, C**). We found that MRGs such as HBB, ALDH1A1, SLC19A3, PC, and COQ8A were highly expressed in normal tissues, whereas genes such as H2BC5, JPT1, STMN1, MRPL12, and H2AX were highly expressed in tumor tissues. We subsequently evaluated the correlation between the expression of Macrophage-related genes and that of other genes. Specifically, we found that MKI67 was positively correlated with genes such as KIF2C, CCNA2, and RACGAP1 but was significantly negatively correlated with genes such as TK2 and PNPLA2 (**Figures 2D-F**). Moreover, we conducted CIBERSORT analysis to evaluate immune cell infiltration in the samples. The results revealed significant differences in immune cell infiltration between breast cancer samples and control samples, highlighting variations in the levels of M0 macrophages, M1 macrophages, T follicular helper (Tfh) cells, regulatory T cells (Tregs), and other types of macrophages (**Figure 2G**).

### 3.2 Consensus clustering of BRCA genes on the basis of the expression of MRGs and functional enrichment between clusters

**Figure 3:**
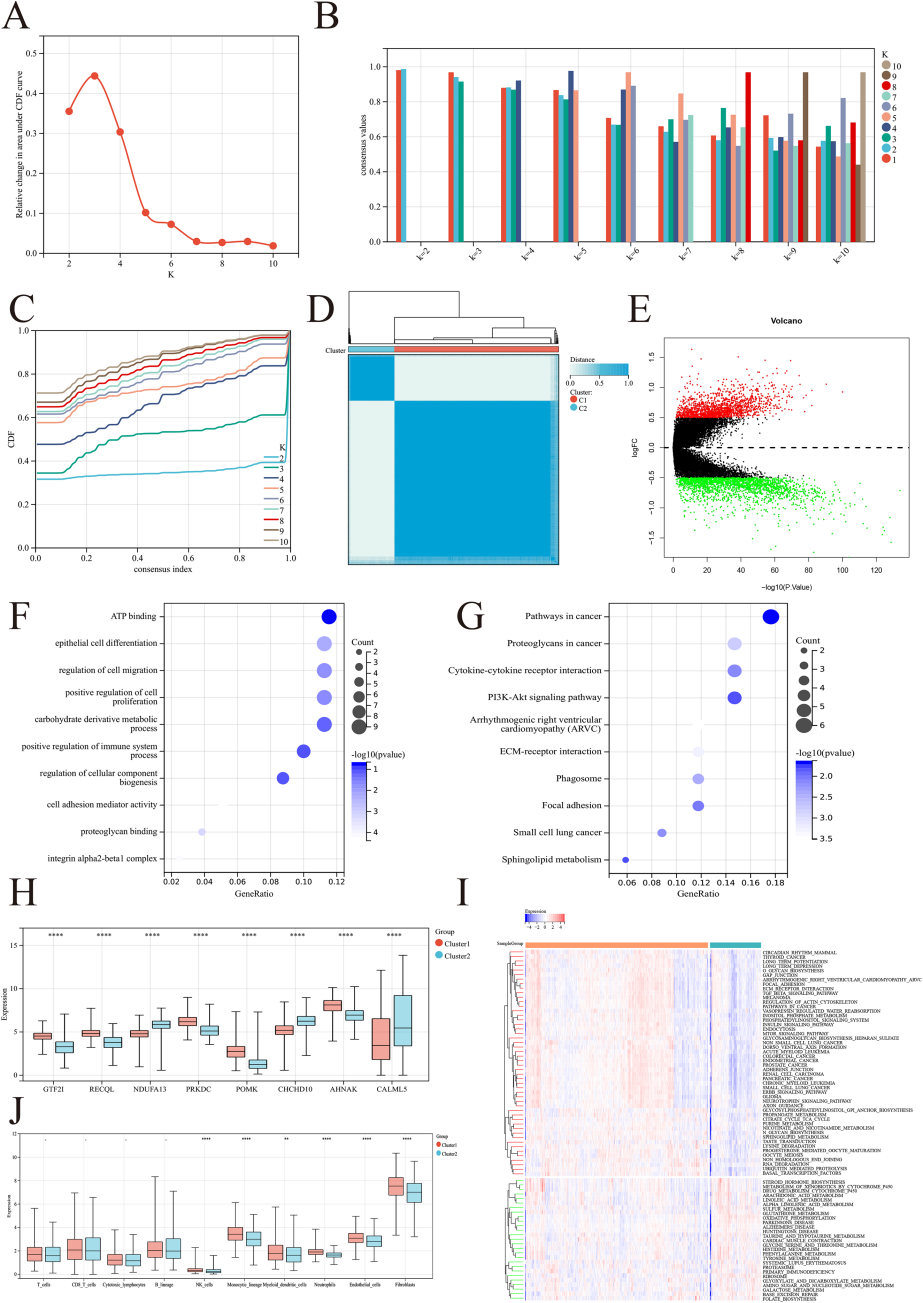
Consensus clustering of BRCA based on MRGs expression and functional enrichment analysis. **(A-D)** Consensus clustering identified optimal cluster number (k=2) based on CDF curve analysis and cluster consistency evaluation, resulting in Cluster 1 (n=853) and Cluster 2 (n=235). **(E)** Volcano plot showing DEGs between clusters (1598 upregulated and 2101 downregulated genes in Cluster 1 vs. Cluster 2). **(F, G)** Functional enrichment analysis of differential genes. **(H)** Expression differences of key MRGs between clusters: Cluster 1 showed elevated expression of GTF2I, RECQL, PRKDC, POMK, and AHNAK, while Cluster 2 exhibited higher levels of NDUFA13, CHCHD10, and CALML5. **(I)** GSEA analysis showed distinct pathway enrichments. **(J)** Immune cell infiltration analysis revealed higher presence of monocytes, NK cells, and dendritic cells in Cluster 1, supporting the GSEA findings. **p<0.01; ***p<0.001; ****p<0.0001.

To examine the features of Macrophage in BRCA, we utilized consensus clustering analysis to categorize the samples on the basis of the expression levels of MRGs. By analyzing the area under the CDF curve and assessing the consistency within groups, we identified the optimal number of clusters as k = 2. This resulted in the classification of the samples into Cluster 1 (n=853) and Cluster 2 (n=235) (**Figures 3A-D**). The DEGs between the two clusters were mapped via a volcano plot, which included 1598 genes whose expression was upregulated and 2101 genes whose expression was downregulated (cluster 1 vs. cluster 2) (**Figure 3E**). GO analysis revealed that the DEGs were enriched primarily in ATP binding, epithelial cell differentiation, cell migration regulation, and positive regulation of immune system processes. Moreover, the KEGG analysis revealed that the DEGs were enriched mainly in pathways related to cancer, proteoglycans in cancer, cytokine–cytokine receptor interactions, and the PI3K–Akt signaling pathway (**Figures 3F, G**). This analysis highlights the significant role of MRGs in tumor growth, metabolism, and immunity. We assessed the differences in the expression of MRGs between the two clusters. Our findings revealed that Cluster 1 displayed elevated expression of GTF2I, RECQL, PRKDC, POMK, and AHNAK, whereas Cluster 2 presented increased levels of NDUFA13, CHCHD10, and CALML5 (**Figure 3H**). These DEGs among clusters may play potential regulatory roles in the Macrophage process of BRCA. To explore the functions of genes from two clusters within the samples, we subsequently performed GSEA to determine the enriched gene sets in each cluster. The results revealed that the pathways significantly enriched in Cluster 1 included intracellular metabolic synthesis and catabolism-related pathways, such as glycan biosynthesis, inositol phosphate metabolism, propanoate metabolism, sphingolipid metabolism, the ubiquitin-mediated proteolysis pathway, and tumor-related pathways, including basal transcription factors, the TGF-β signaling pathway, and the mTOR signaling pathway. In contrast, a variety of metabolite synthesis processes, including steroid hormone biosynthesis, metabolism of xenobiotics by cytochrome P450, glyoxylate metabolism, linoresinol metabolism, glutathione metabolism, histidine metabolism, and dicarboxylate metabolism, were significantly enriched in cluster 2. Notably, we found enrichment of the primary immunodeficiency pathway in cluster 2, implying a more suppressive immune microenvironment (**Figure 3I**). We conducted additional analysis of immune cell infiltration across different clusters. We observed that cluster 1 exhibited a greater presence of immune cells, including monocytes, NK cells, and dendritic cells (**Figure 3J**). This finding partly supports the earlier results from the GSEA.

### 3.3 Establishing a risk model related to MRGs in BRCA patients

To better understand the relationship between MRGs and the prognosis of BRCA patients, we obtained DEGs between the tumor tissues. We matched normal tissue, as well as between Cluster 1 and Cluster 2, and a total of 112 genes were included in further studies (**Supplementary Figure 1A**). Additional screening of intersecting genes with prognostic value revealed that the expression of DST, SORBS1, SAA2-SAA4, KRT14, KRT17, MUCL1, IGHA2, and SLPI was positively correlated with a better prognosis. In contrast, HAGHL and RHPN1 might be potential oncogenes in BRCA (**Supplementary Figure 1B**). We then performed LASSO regression analysis and identified three key genes, HAGHL, SLPI, and HSPB6, which formed the basis of a predictive model known as the MRGs score model. The risk score represented by the model was calculated on the basis of the expression of the above three key genes: risk score = 0.120888647718605 * HAGHL - 0.100980394404445 * HSPB6 - 0.0720652798459613 * SLPI. We developed a prognostic model that integrates key genes, effectively stratifying BRCA patients by risk. Patients were classified into high- and low-risk groups on the basis of the median risk score, with K–M analysis revealing significantly better survival in the low-risk group (p=0.021) (**Supplementary Figure 1C*****)***. The expression of SLPI and HSPB6 was negatively correlated with the risk score, whereas HAGHL was positively correlated, indicating a worsening prognosis as the risk increased (**Supplementary Figures 1D-F**). Multivariate analysis confirmed that the risk score was an independent prognostic factor for BRCA (p<0.001) (**Supplementary Figures 1G, H**). The predictive model demonstrated good performance, achieving AUC values of 0.755, 0.733, and 0.668 for predicting OS at 1, 5, and 10 years, respectively (**Supplementary Figures 2A, B**). We developed a nomogram on the basis of this model and patient clinical information (**Supplementary Figure 2C*****).*** The calibration curve indicated that the nomogram provides accurate predictions for both 3-year and 5-year OS ***(*****Supplementary Figure 2D**). Furthermore, the nomogram demonstrated enhanced predictive ability, with AUC values of 0.662, 0.638, and 0.652 for OS prediction at 1, 3, and 5 years, respectively (**Supplementary Figure 2E**). K–M survival analysis further demonstrated the precise prognostic predictive ability of the nomogram in BRCA patients (p<0.001) (**Supplementary Figure 2F**).

### 3.4 Mutational and immune infiltration characteristics of BRCA subtypes

**Figure 4:**
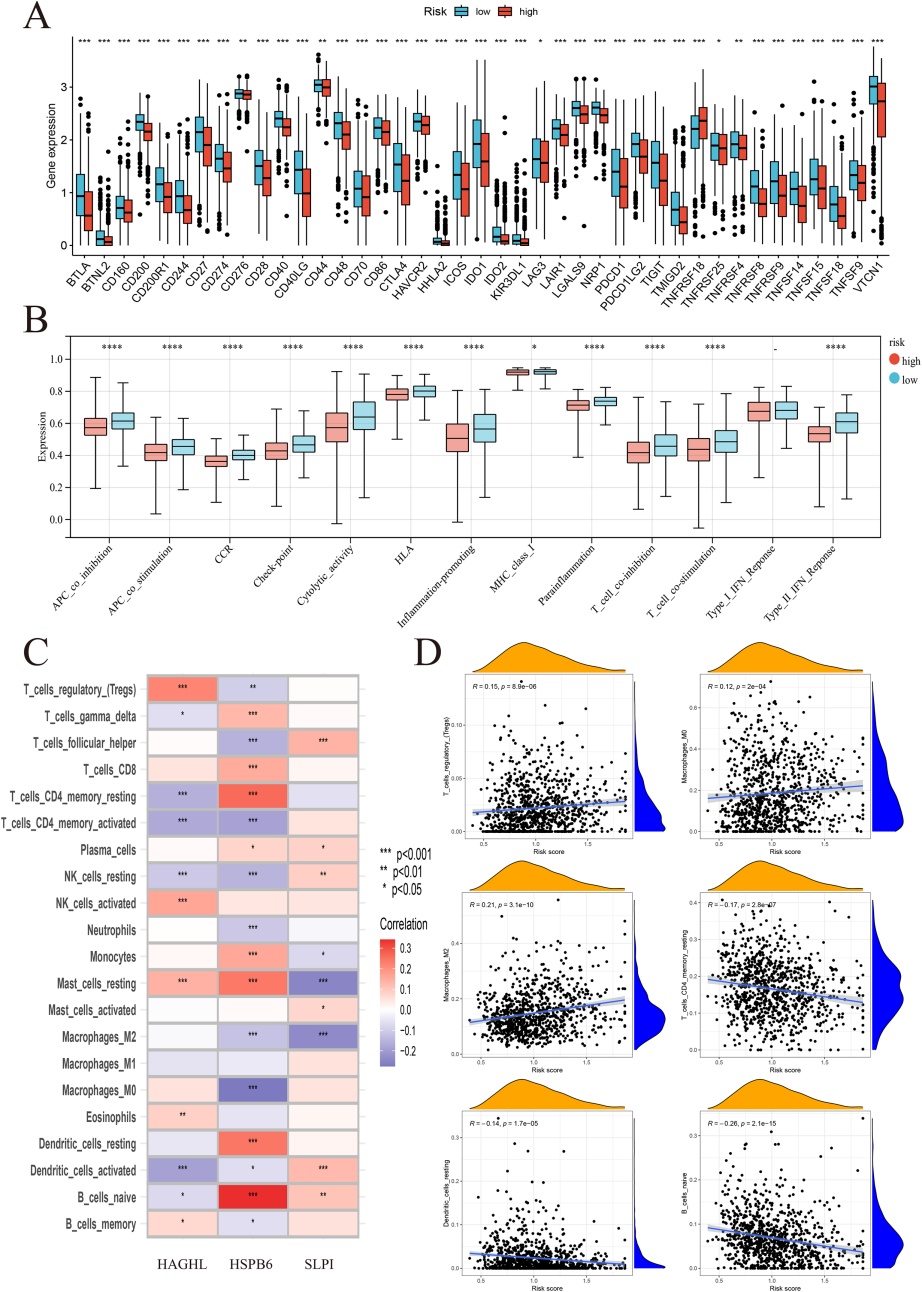
Immune checkpoint expression and immune infiltration characteristics of BRCA subtypes. **(A)** Classical immune checkpoint markers (PD-1, PD-L1, CTLA-4) were expressed at higher levels in the low-risk group. **(B)** Immune function analysis revealed enrichment of multiple immune processes in the low-risk group, suggesting increased immune activity and potential sensitivity to immunotherapy. **(C)** Correlation analysis showed distinct associations between key genes and immune cell infiltration. **(D)** The risk score was associated with specific immune cell populations: positively correlated with Tregs, M0 macrophages, and M2 macrophages; negatively correlated with memory resting CD4+ T cells, resting dendritic cells, and naïve B cells. *p<0.05; **p<0.01; ***p<0.001; ****p<0.000.

We stratified patients by risk score and analyzed mutation profiles and found significant differences between the high- and low-risk groups, with PIK3CA, TP53, GATA3, TTN, and MUC16 showing distinct mutation frequencies (**Supplementary Figures 3A, B**). High-risk tumors presented increased numbers of Tregs, M0/M2 macrophages, and resting mast cells, whereas low-risk tumors presented increased numbers of naïve B cells, CD8+ T cells, and M1 macrophages (**Supplementary Figure 3C**). MCP analysis confirmed greater T cell, cytotoxic lymphocyte, B cell, and myeloid cell infiltration in low-risk tumors than in high-risk tumors (**Supplementary Figure 3D**). ESTIMATE analysis further revealed higher stromal/immune scores and lower tumor purity in low-risk patients than in high-risk patients (**Supplementary Figures 3E, F**). Our analysis of various immune checkpoints revealed that classical markers such as PD-1, PD-L1, and CTLA-4 were expressed at relatively high levels in the low-risk group (**Figure 4A**). Furthermore, immune function analysis revealed that several immune processes, including APC coinhibition, APC costimulation, CCR, checkpoint activity, cytolytic activity, and HLA, were enriched in the low-risk group (**Figure 4B**). The results of our analysis suggested an increased likelihood of immune escape in patients in the high-risk group, which may lead to a decrease in the effectiveness of ICI therapy. Conversely, the low-risk group may be more sensitive to immunotherapy. To better understand the potential link between key genes and the degree of tumor immune infiltration, as well as to conduct follow-up studies, we used correlation analysis and found that key genes were associated with multiple immune cell infiltrates. For instance, higher HAGHL expression was linked to increased infiltration of Tregs and activated NK cells, but reduced presence of activated dendritic cells. In parallel, elevated HSPB6 levels were associated with greater numbers of naïve B cells and memory resting CD^+^ T cells, yet lower counts of M0 macrophages (**Figure 4C**). These results suggest avenues for further investigation into the regulatory roles of key genes within the TME. Additionally, we found that the risk score was associated with several immune cells; specifically, it was positively correlated with Tregs, M0 macrophages, and M2 macrophages but negatively correlated with memory resting CD4+ T cells, resting dendritic cells, and naïve B cells (**Figure 4D**).

### 3.5 Excellent predictive performance of the MRGs model for prognosis and immunotherapy effectiveness in BRCA patients

**Figure 5:**
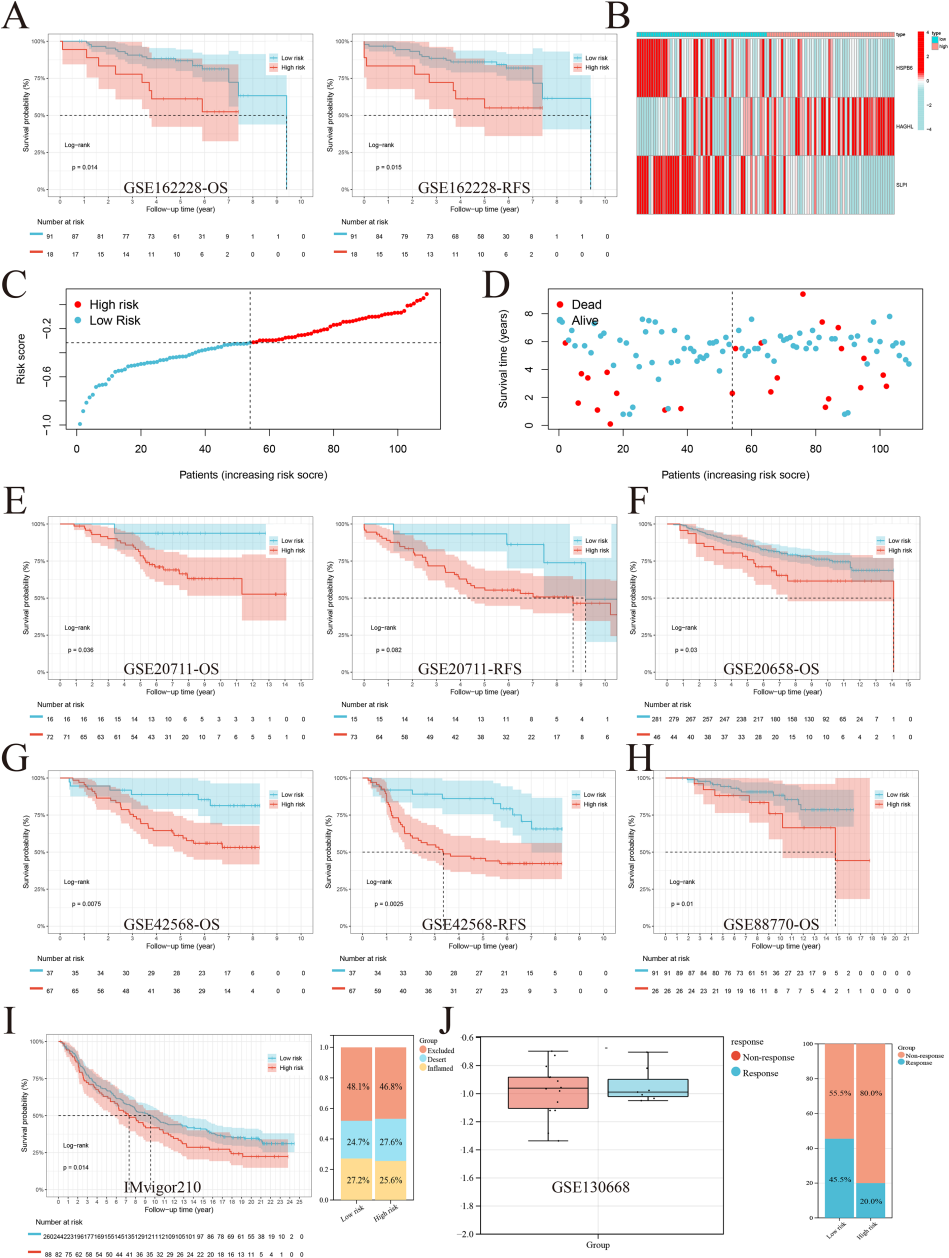
Validation of the MRGs model’s prognostic and immunotherapy-predictive performance across multiple datasets. **(A)** In the GSE162228 external cohort, low-risk patients exhibited significantly better OS and RFS. **(B-D)** Consistent with the training set, with low-risk patients having better prognosis. **(E-H)** The model’s prognostic performance was validated in multiple independent datasets. **(I)** In the Imvigor210 immunotherapy cohort, low-risk patients showed better prognosis and immune-inflamed phenotypes, suggesting higher immunotherapy sensitivity. **(J)** In the GSE103668 neoadjuvant therapy cohort, low-risk patients had a higher response rate (45.5%) compared to high-risk patients (20%), though the difference between responder and non-responder groups did not reach statistical significance.

After demonstrating the reliability of the risk score in predicting prognosis and immune microenvironment differences in BRCA patients, we further validated the model’s efficacy via external data. In the GSE162228 dataset, patients with a low risk score had better OS (p = 0.014) and RFS (p = 0.015) (**Figure 5A**). Similar to the training set, patients with lower expression of HSPB6 and SLPI and higher expression of HAGHL had progressively higher risk scores, implying a worse prognosis and a greater risk of death (**Figures 5B-D**). Similarly, we found that patients in the low-risk group had better OS in the GSE20658 and GSE88770 datasets and better OS and RFS in the GSE20711 and GSE42568 datasets (**Figures 5E-H**). We next assessed the model’s predictive performance in the IMvigor210 cohort for clinical immunotherapy response. The findings indicate that low−risk patients are likely to show greater sensitivity to immunotherapy and enjoy improved outcomes. Moreover, a reduced risk score was linked to an immune−inflamed tumor phenotype (**Figure 5I**). These findings highlight the potential role of the risk score as a predictive biomarker of immunotherapy response. The GSE103668 dataset includes gene expression data from 21 TNBC samples treated with cisplatin and bevacizumab in neoadjuvant therapy. We found that the nonresponsive treatment group had a higher risk score than did the responsive treatment group. Unfortunately, we did not observe a significant difference, probably due to an insufficient sample size. We found a response rate of 45.5% in the low-risk group and 20% in the high-risk group (**Figure 5J**), which somewhat validates the ability of the risk score to serve as a predictive tool for immunotherapy.

### 3.6 Clinical relevance and pathway enrichment analysis

**Figure 6:**
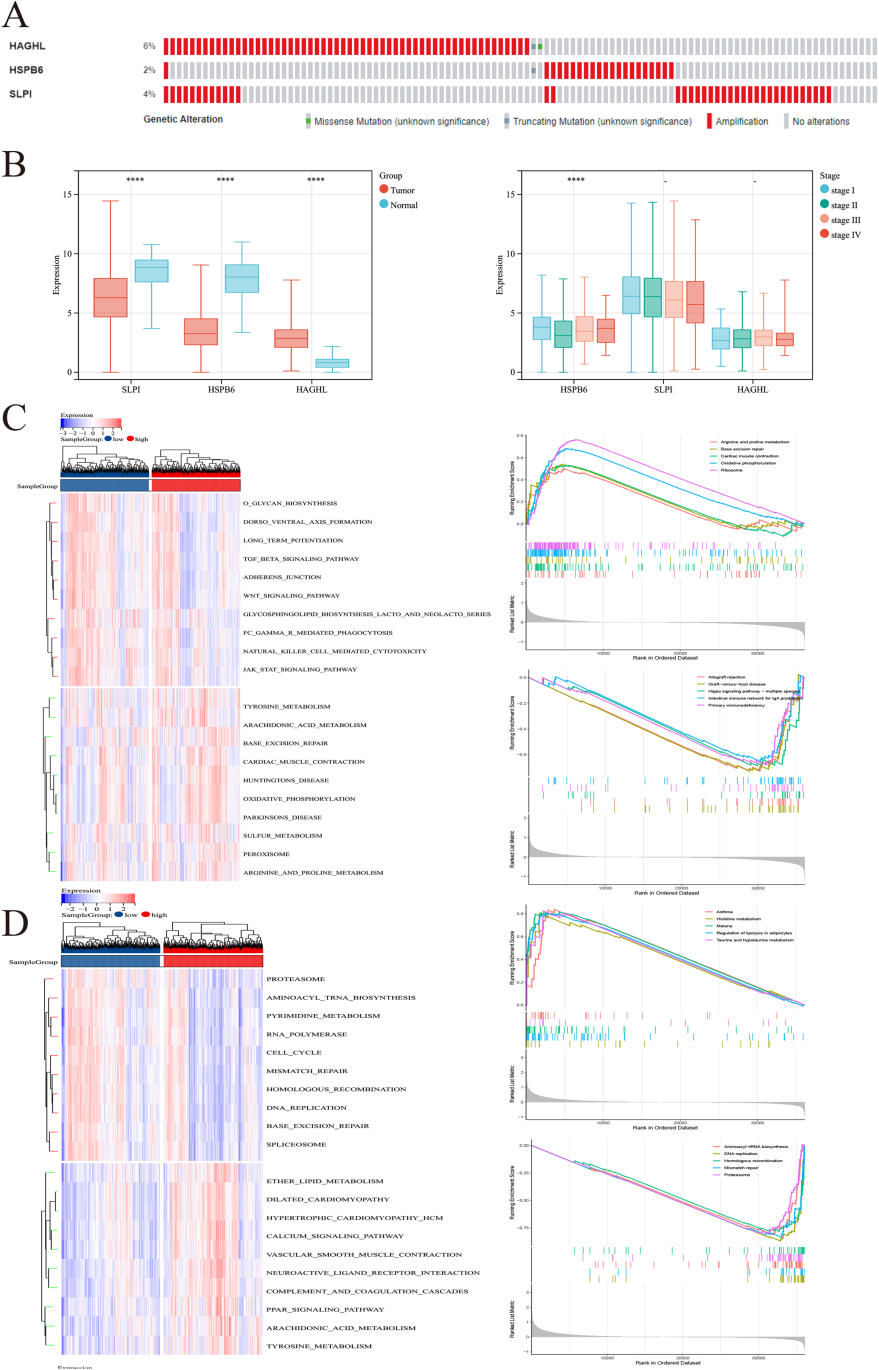
Clinical relevance and pathway enrichment analysis of key MRGs in BRCA. **(A)** Oncoprint profiles showing somatic mutation frequencies of HAGHL (6%), HSPB6 (2%), and SLPI (4%) in BRCA (cBioPortal database). **(B)** Correlation between gene expression and tumor stage. **(C)** Correlation between key gene expression and TMB. **(D)** GSVA analysis showing pathway enrichment patterns for each key gene. ****p<0.0001.

Our results revealed three key genes (HAGHL, HSPB6, and SLPI), and we subsequently attempted to investigate the biological characterization of these three genes in BRCA. Initially, we obtained oncoprint profiles via the cBioPortal database. The frequencies of somatic mutations observed for HAGHL, HSPB6, and SLPI were 6%, 2%, and 4%, respectively (**Figure 6A**). On the basis of the clinical data of the TCGA-BRCA cohort, HSPB6 and SLPI were expressed at low levels, whereas HAGHL was highly expressed in tumors. HSPB6 expression decreased with advancing tumor stage. Similarly, SLPI expression tended to decrease with increasing tumor stage, and HAGHL tended to correlate with advanced tumor stage (**Figure 6B**). Most likely, because of the small sample size of patients at Stage IV, we did not obtain statistically significant differences. Moreover, expression of the three key genes was linked to tumor mutational burden (TMB): high HSPB6 and HAGHL levels corresponded to lower TMB, whereas SLPI showed an inverse association (**Figure 6C**). We conducted a more in-depth analysis of the signaling pathways related to the three crucial genes to explore their possible molecular mechanisms. GSVA revealed that when HAGHL was highly expressed, it primarily activated various metabolic processes, including those for tyrosine, arachidonic acid, and proline, as well as cellular energy metabolism processes such as oxidative phosphorylation. When HSPB6 is expressed at low levels, biological processes such as the cell cycle, mismatch repair, and homologous recombination are activated. In contrast, lipid metabolism processes such as ether lipid and arachidonic acid biosynthesis, the calcium signaling pathway, the PPAR signaling pathway, etc., were suppressed (**Figure 6D**). Furthermore, when SLPI expression was low, the activated pathways involved selenoamino acid metabolism, aminoacyl tRNA biosynthesis, and ubiquitin-mediated proteolysis. Conversely, the pathways that were inhibited included xenobiotic metabolism by cytochrome P450, interactions between neuroactive ligands and receptors, and cytokine receptor interactions (**Supplementary Figure 4**). The results of GSEA revealed that HAGHL had a significant role in arginine and proline metabolism, ribosome, and oxidative phosphorylation; HSPB6 was enriched in histone metabolism and the regulation of lipolysis in adipocytes; and SLPI was enriched in allograft rejection, primary immunodeficiency, etc. These findings suggest that the three key genes can influence BRCA by regulating the synthesis of multiple intracellular metabolites and multiple tumor-related signaling pathways.

### 3.7 Exploring the expression and distribution of model genes at the single-cell level

**Figure 7:**
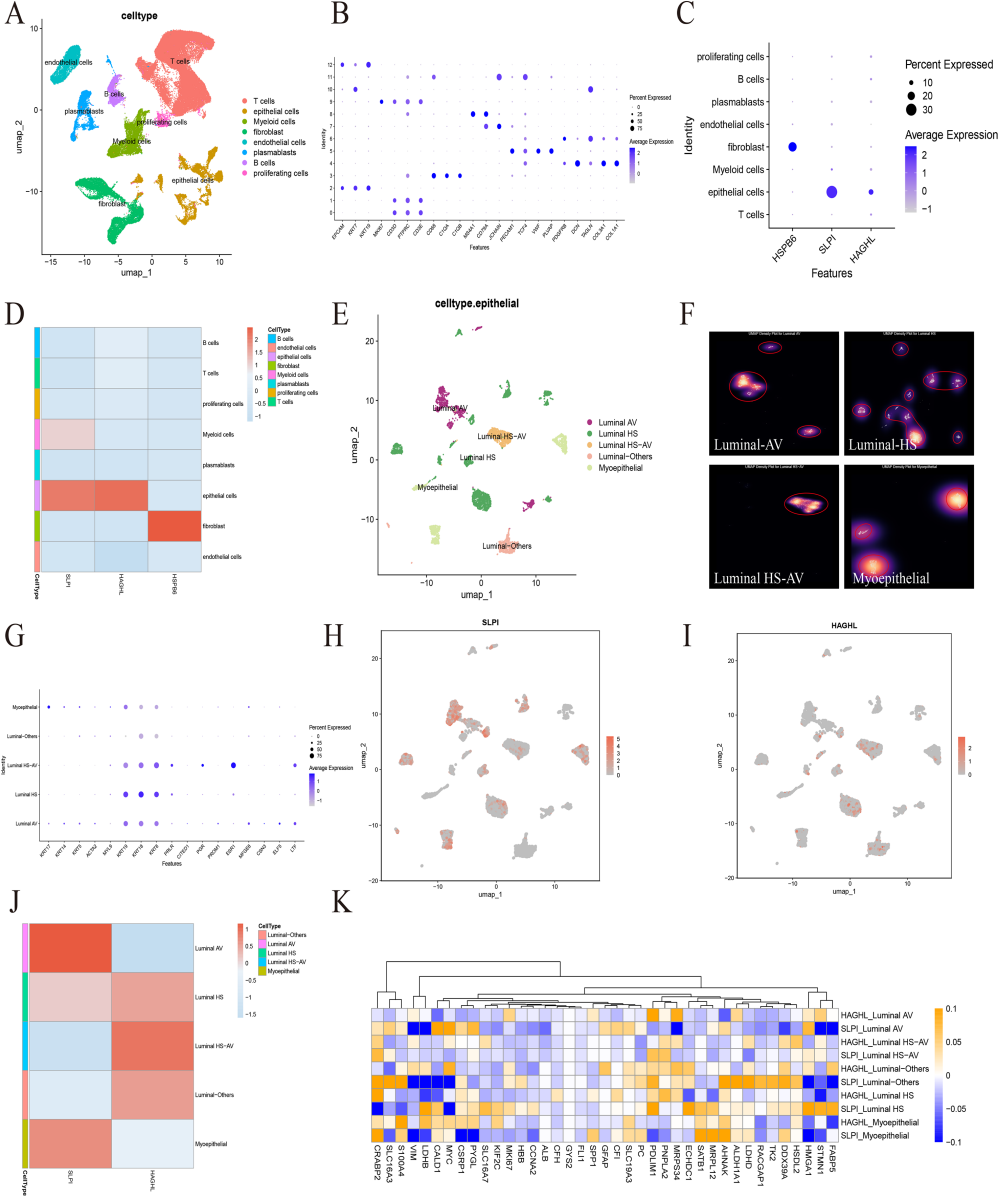
Single-cell RNA sequencing analysis of key MRGs expression patterns in BRCA. **(A)** t-SNE plot showing the clustering of 26 primary BRCA tumors into eight major cell types identified through scRNA-Seq analysis. **(B)** Bubble plot displaying marker genes for each of the eight cell clusters (T cells, epithelial cells, myeloid cells, fibroblasts, endothelial cells, plasmablasts, B cells, and proliferating cells). **(C, D)** Expression distribution of key genes across cell types. **(E, F)** Further classification of epithelial cells into five subtypes: Luminal AV, Luminal HS, Luminal HS-AV, myoepithelial, and Luminal others. **(G)** Bubble plot showing marker genes for the five epithelial cell subtypes. **(H-J)** Differential expression patterns of SLPI and HAGHL within epithelial subtypes. **(K)** Correlation analysis revealing: In Luminal AV: SLPI positively correlates with CALD1, MYC, HMGA1; negatively correlates with VIM, LDHB, STMN1, FABP5.

To characterize the molecular profile of key genes more profoundly in BRCA, we used single-cell sequencing data for subsequent analysis. The GSE176078 dataset from the GEO database utilized single-cell RNA sequencing (scRNA-Seq) through Chromium technology from 10X Genomics. Twenty-six primary BRCA tumors representing three principal clinical subtypes were analyzed: 11 ER+, 5 HER2+, and 10 TNBC subtypes. After preliminary processing of the data, we classified all the cells into eight clusters, including T cells, epithelial cells, myeloid cells, fibroblasts, endothelial cells, plasmablasts, B cells, and proliferating cells (**Figure 7A**). A bubble plot was used to represent the marker genes for each cell type (**Figure 7B**). We found that HSPB6 was highly expressed mainly in fibroblasts, whereas SLPI and HAGHL were coexpressed in epithelial cells (**Figures 7C, D**). The latter attracted our interest, and we extracted epithelial cells for subsequent analysis. We categorized the epithelial cells into five types, namely, luminal AV, luminal HS, luminal HS-AV, myoepithelial, and luminal others, according to Brugge et al. (**Figures 7E, F**). The bubble plot shows the marker genes for the epithelial cell types (**Figure 7G**). We found that SLPI was highly expressed mainly in Luminal AV, whereas HAGHL was highly expressed mainly in Luminal HS-AV (**Figures 7H-J**). We subsequently analyzed the correlation of SLPI and HAGHL with the expression of MRGs in epithelial cell subtypes and found that in the luminal AV subtype, the expression of SLPI was significantly positively correlated with CALD1, MYC, and HMGA1, whereas a significant negative correlation was observed with VIM, LDHB, STMN1, and FABP5. In the Luminal HS-AV subtype, the expression of HAGHL was negatively correlated with SLC16A3, PC, and HMGA1 and positively correlated with CRABP2, HSDL2, etc. (**Figure 7K**). These results suggest that SLPI and HAGHL may have a potential connection with the process of Macrophage in epithelial cells and provide a good idea for subsequent studies targeting the mechanism of Macrophage in BRCA.

### 3.8 Analysis of interactions between epithelial cells with high HAGHL expression and other cells

**Figure 8:**
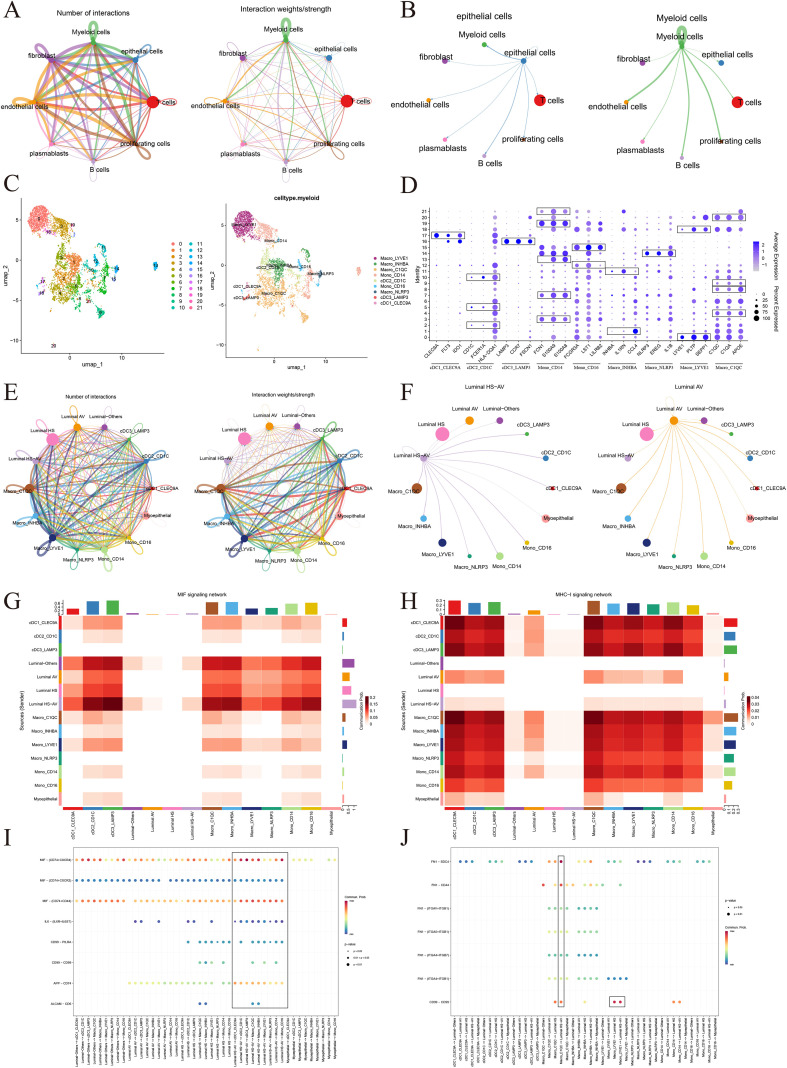
Cell-cell communication analysis between HAGHL-high Luminal HS-AV epithelial cells and other cell types in BRCA microenvironment. **(A, B)** CellChat analysis revealing extensive intercellular communication networks between epithelial cells and other cell types. **(C)** Classification of myeloid cells into nine distinct subpopulations. **(D)** Bubble plots displaying marker genes for each myeloid cell subpopulation. **(E)** Comparative analysis showing that epithelial cells exhibit significantly weaker communication with non-myeloid cells compared to myeloid cells. **(F)** Specific communication patterns between epithelial subtypes and myeloid cells. **(G, H)** Differential signaling pathway enrichment in epithelial-myeloid interactions. **(I, J)** Detailed ligand-receptor interaction pathways.

Next, we aimed to determine the relationships between luminal HS-AV epithelial cells with high HAGHL expression and luminal AV epithelial cells with high SLPI expression and other cells in the BRCA microenvironment. We then utilized CellChat, a tool for analyzing cell–cell communication from scRNA–seq data, via the ligand–receptor interaction database. Our results revealed interactions between epithelial cells and other types of cells. Combined with the number of interactions and interaction weights, we found that epithelial cells had a more significant intercellular communication rate with myeloid cells (**Figures 8A, B**). Zhang et al. subdivided myeloid cells into nine subgroups: Macro_LYVE1, Macro_INHBA, Macro_C1QC, Macro_NLRP3, Mono_CD14, Mono_CD16, cDC1_CLEC9A, cDC2_CD1C, and cDC3_LAMP3 (**Figure 8C**). Bubble plots demonstrate the marker genes for each myeloid cell subpopulation (**Figure 8D**). We found that the chance of intercellular communication between epithelial cells and other cells was significantly lower than that between myeloid cells (**Figure 8E**); therefore, we targeted luminal HS-AV epithelial cells, which have high expression of HAGHL and strongly associated with cDC2_CD1C, cDC3_LAMP3, Macro_INHBA, Macro_C1QC, Mono_CD14, and Mono_CD16. Similarly, for luminal AV epithelial cells, we detected stronger associations with Macro_C1QC, Macro_LYVE1, cDC2_CD1C, and Mono_CD16 (**Figure 8F**). In addition, by screening the possible signaling pathways involved in intercellular communication, we found that MIF signaling, such as the intercellular communication between Luminal HS-AV epithelial cells and myeloid cells, was significantly enhanced between Luminal HS-AV epithelial cells and cDC3_LAMP3 (**Figure 8G**). In contrast, for luminal AV epithelial cells, MHC-1 signaling was enhanced in communication with myeloid cells (**Figure 8H**). Finally, we analyzed the ligand–receptor pathways between luminal HS-AV epithelial cells and myeloid cells and found that they interact with each other primarily through the MIF (macrophage migration inhibitory factor)- (CD74+CXCR4), MIF- (CD74+CD44), and APP-CD74 ligand pathways. Among them, the interaction of luminal HS-AV epithelial cells with cDC3_LAMP3 cells occurred mainly through the MIF (CD74+CXCR4) ligand pathway, and the interaction of luminal HS-AV cells with Macro_C1QC cells occurred mainly through the MIF (CD74+CD44) and FN1-SDC4 ligand pathways (**Figures 8I, J**). These findings suggest that luminal HS-AV epithelial cells may interact with a variety of myeloid cells through this ligand–receptor pathway, thereby affecting the progression of BRCA, and may be highly relevant to the process of Macrophage in the microenvironment.

### 3.9 HAGHL knockdown inhibits the activity of BRCA cells

**Figure 9:**
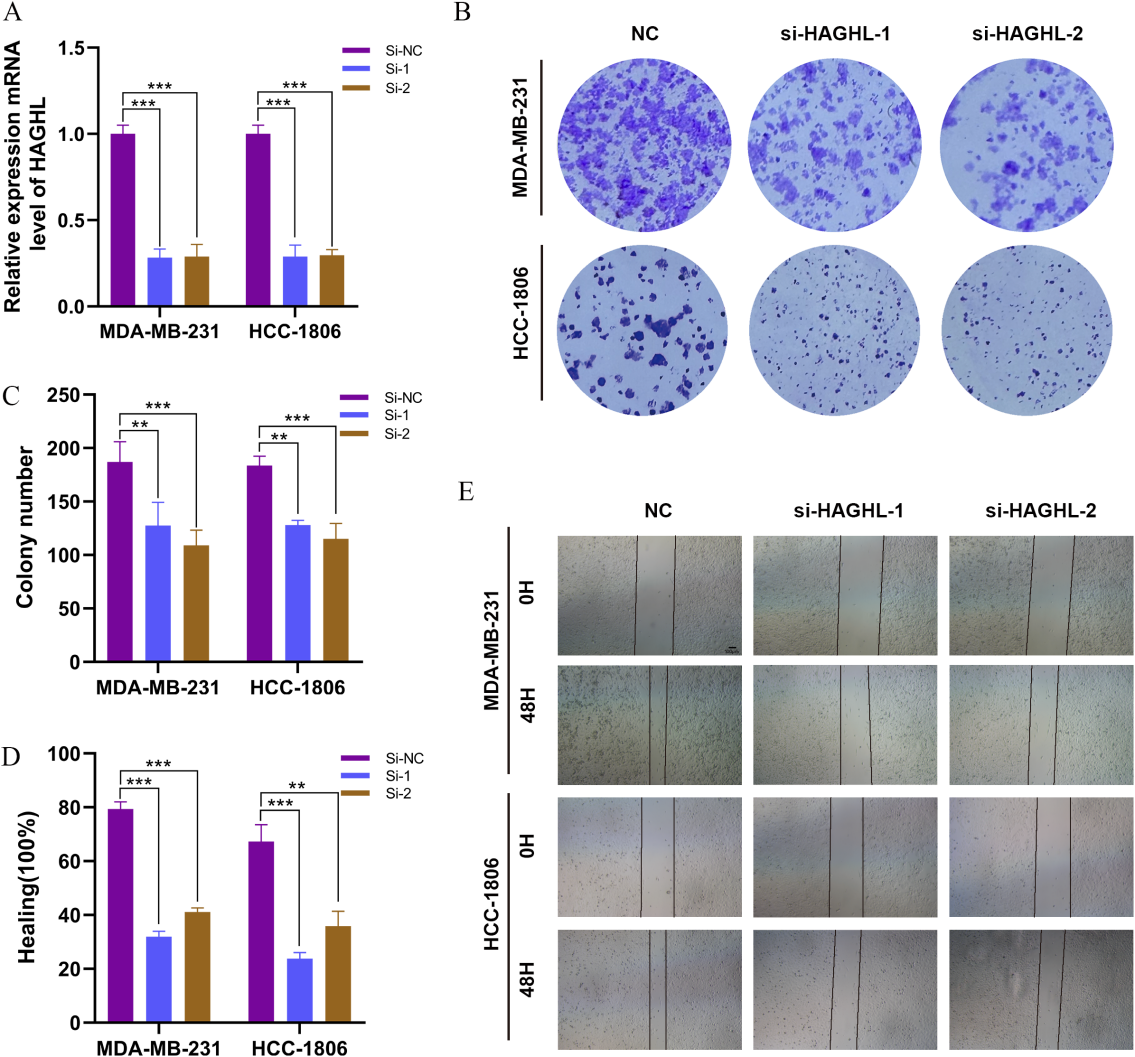
HAGHL knockdown inhibits proliferation, and migration of BRCA cells. **(A)** Validation of HAGHL knockdown efficiency in MDA−MB−231 and HCC1806 cells by RT−qPCR. (**B, C**) Representative images of colony formation assays showing reduced colony numbers and sizes in HAGHL-knockdown MDA-MB-231 and HCC1806 cells compared to controls. (**D, E**) Wound healing assay images showing decreased migration of HAGHL-knockdown cells compared to control cells. (*p<0.05, **p<0.01, ***p<0.001).

In order to further investigate the effect of HAGHL on the proliferation ability of BRCA cells, we knocked down the HAGHL gene in MDA-MB-231 and HCC1806 cells (**Figure 9A**), and then conducted colony formation experiments (**Figure 9B**). The results revealed that knocking down the HAGHL gene led to a reduction in both the number and size of colonies formed by the two BRCA cell lines MDA-MB-231 and HCC1806 (**Figure 9C**). In the wound healing assay, we observed a significant decrease in the migration of the two HAGHL-knockdown cell lines compared with that of the control cells (**Figure 9D**). The bar chart analysis indicated that the differences between the two groups were statistically significant (**Figure 9E**).

## 4 Discussion

In recent years, clinical practice and translational research on BRCA immunotherapy have made significant milestone advancements, particularly highlighting ICIs that target PD-1 and PD-L1 (39–41). In the treatment of different molecular subtypes of BRCA, the combination of ICIs with traditional therapies remains the primary strategy for immunotherapy (13). However, ICI treatment still faces challenges such as complex resistance mechanisms, therapeutic heterogeneity, and the management of immune-related adverse events (42, 43). Furthermore, there is a need to optimize patient stratification management and explore more precise combination treatment strategies to ensure long-term survival benefits for patients with BRCA mutations (44). Here, our research reveals the molecular characteristics and prognostic significance of MRGs in BRCA, emphasizing the importance of macrophage as a predictive factor for the efficacy of immunotherapy. This study also highlights the tremendous potential of macrophage-related features in advancing precise and personalized immunotherapy strategies.

Dysregulated MRGs not only affect cancer cell plasticity and promote more invasive phenotypes but also play crucial roles in immune cell functions (45). For example, Macrophage of lysines 120 (K120) and 139 (K139) in the DNA-binding domain of p53, which is mediated by alanyl-tRNA synthetase (AARS1), contributes to tumor progression (46). Lowering lactate levels through LDH inhibitors combined with anti-PD-1 therapy has been shown to elicit a stronger antitumor response than PD-1 blockade alone (47). Studies have further illuminated the role of lactate-induced macrophage in immune suppression within the TME. For example, macrophage at K70 of APCO2 is upregulated in non-small cell lung cancer, driving metastasis. APCO2-K70 macrophage can enhance immunotherapy resistance by increasing the ratios of Treg and CD8+ T cells (48). Additionally, lactate-induced H3K9 macrophage is enriched in the IL11 gene in tumor cells, where IL-11 promotes CD8+ T-cell dysfunction via the JAK2/STAT3 signaling pathway (49). These findings suggest that macrophage plays a critical role in shaping the immune-suppressive TME.

HAGHL (hydroxyacylglutathione hydrolase) is a metabolic enzyme encoded by the nuclear genome, is located in the cytoplasm, and is involved primarily in the glutathione metabolism pathway (50). It catalyzes the hydrolysis of S-lactoylglutathione into reduced glutathione (GSH) and lactate (51). Our study is the first to demonstrate that the key Macrophage gene HAGHL acts as an oncogenic factor in BRCA, with its knockdown capable of inhibiting the proliferation and invasion of BRCA cells.

After stratifying patients by risk score, we evaluated the differences in the immune microenvironment across risk groups (52). We found that patients in the low-risk group had better prognoses, characterized by higher levels of immune cell infiltration and more robust immune function, suggesting that this subset of the population may be more responsive to immunotherapy. This finding was validated in the IMvigor210 cohort and the BRCA immunotherapy cohort. This MRGs classification provides valuable insights into BRCA treatment, particularly regarding the use of immunotherapy. Additionally, our study focused on several core genes, whose expression levels correlated with immune cell infiltration and tumor mutational burden. Notably, elevated HAGHL expression was associated with increased infiltration of Tregs and activated NK cells, but reduced levels of activated dendritic cells. On the other hand, HSPB6 was positively associated with naïve B cells and memory resting CD4+ T cells but negatively correlated with M0 macrophages. Through cellChat analysis, we identified potential interactions between the luminal HS-AV epithelial subgroup, which highly expresses HAGHL, and various macrophages and dendritic cells via the MIF pathway. The MIF-CD74 axis is involved in impairing the antitumor activity of immune cells, promoting tumor proliferation and immune evasion, and is a potential therapeutic target. Although our initial functional experiments have confirmed the oncogenic role of HAGHL in BRCA, the specific molecular mechanism by which it regulates the TME, especially its association with the known immunosuppressive pathway MIF-CD74 axis, is still mainly based on co-expression analysis and pathway enrichment results from bioinformatics, and lacks direct experimental evidence.

Given the limited efficacy of traditional molecular subtyping in predicting individual responses to adjuvant therapy in BRCA, there is an urgent need for more precise biomarkers (53). Therefore, we aimed to characterize the molecular expression patterns of MRGs and develop a robust prognostic model via multiomics approaches. We therefore developed a novel MRG-based classifier that transcends conventional subtyping. This classifier directly addresses the need for better patient stratification by identifying distinct immune-microenvironment subtypes, thereby providing a actionable framework for tailoring immunotherapy strategies to individual patients and ultimately improving clinical outcomes.

This study is mainly based on the indirect evidence from the correlation analysis of the database. The effectiveness and safety of this strategy lack direct preclinical pharmacological validation. In the future, it is necessary to use the BRCA cell line with high expression of HAGHL in a humanized mouse model to directly compare the efficacy differences between “chemotherapy combined with anti-PD-1 antibody” and single therapy, thereby providing a more solid experimental basis for the design of subsequent clinical trials (54).

## 5 Conclusions

This study systematically elucidated the crucial role of MRGs in the immune microenvironment and prognosis of breast cancer, and for the first time discovered that the metabolic enzyme HAGHL is a new oncogenic factor. Its high expression promotes an immunosuppressive microenvironment through the MIF-CD74 axis. Based on this, the MRGs prognosis model constructed can effectively identify patients with the “immune hot” phenotype who are more sensitive to immunotherapy. Therefore, we proposed a new combined treatment strategy of “chemotherapy + immune checkpoint inhibitors” for patients with high expression of HAGHL, providing a precise diagnosis and treatment direction for overcoming the differences in the efficacy of BRCA immunotherapy.

## Data Availability

The datasets presented in this study can be found in online repositories. The names of the repository/repositories and accession number(s) can be found in the article/**Supplementary Material**.
