# Supplementary material for: Macrophage-associated prognostic modeling uncovers immunotherapy response mechanisms and defines HAGHL as a novel oncogenic driver in breast cancer
Source: Front Immunol. 2026 Mar 26;17:1776875. doi: 10.3389/fimmu.2026.1776875 (PMC13062333; doi:10.3389/fimmu.2026.1776875)
Supplement: Supplementary file 1 [file DataSheet1.docx]

Supplementary Figures


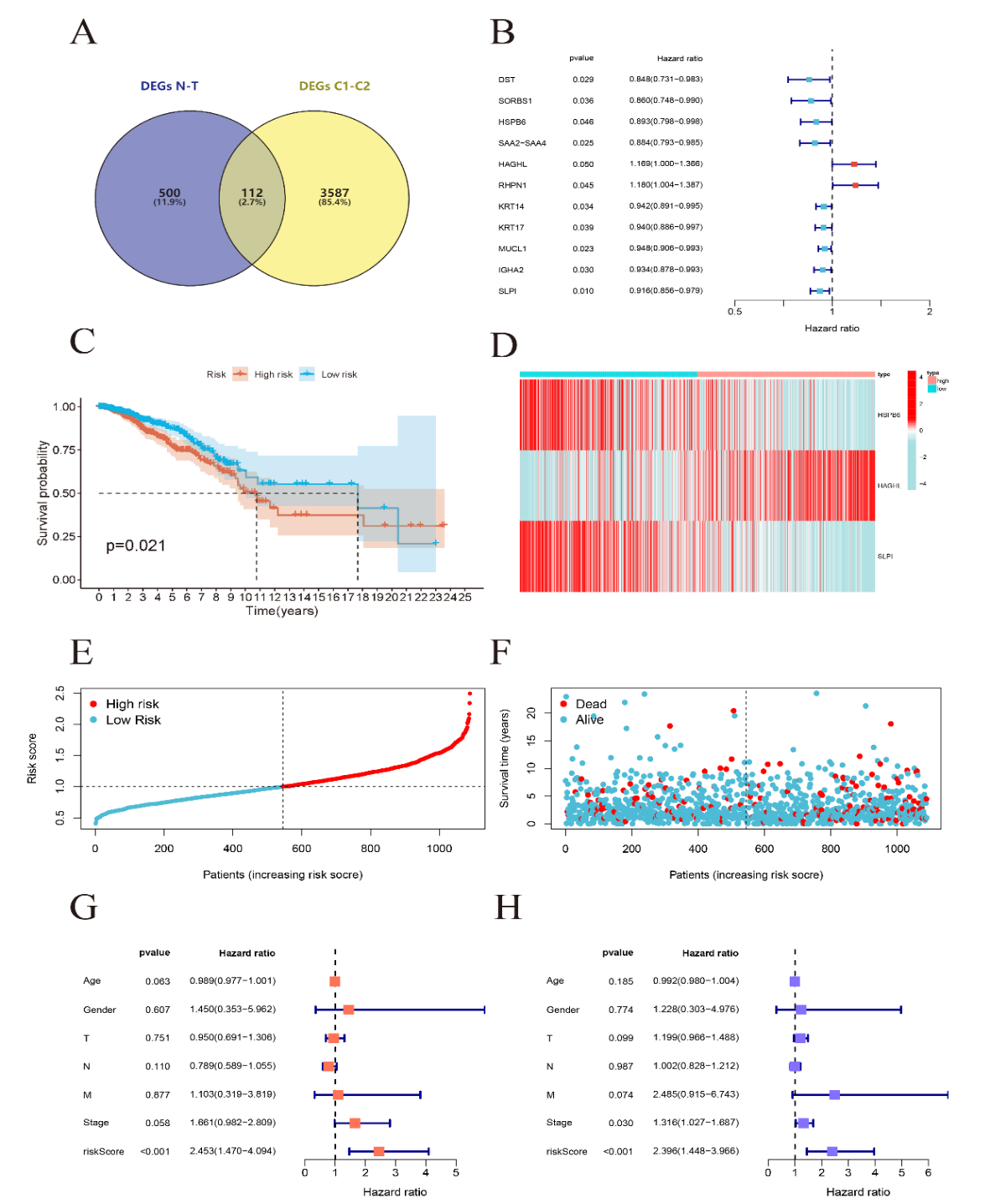


​**​Supplementary Figure 1. Development of the MRGs risk model in BRCA.​**​ (**A**) Identification of 112 DEGs from tumor vs. normal and Cluster1 vs. Cluster2 comparisons for model construction. (**B**) Prognostic screening revealed: Favorable prognostic markers: DST, SORBS1, SAA2-SAA4, KRT14, KRT17, MUCL1, IGHA2, SLPI, Potential oncogenes: HAGHL, RHPN1 (**C**) K-M survival analysis demonstrating significantly better survival in low-risk patients (p=0.021). (**D-F**) Correlation analysis showing: Negative correlation between risk score and SLPI/HSPB6 expression, Positive correlation between risk score and HAGHL expression. (**G-H**) Multivariate Cox regression analysis confirming the risk score as an independent prognostic factor (p<0.001).


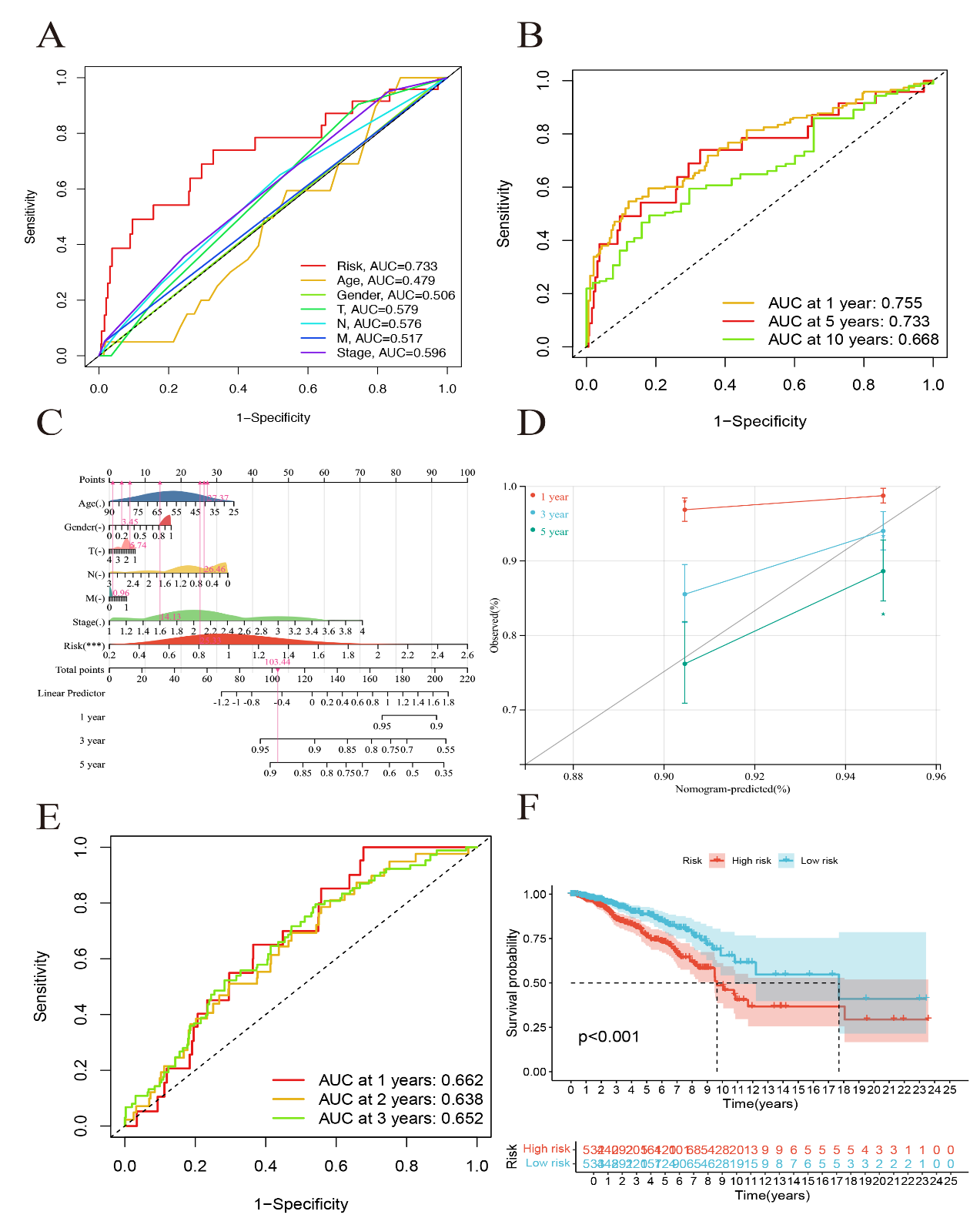


**Supplementary Figure 2. Validation of a MRGs risk model for BRCA prognosis.​**​ (**A-B**) The MRG score model, based on HAGHL, SLPI, and HSPB6 expression, demonstrated good prognostic performance with AUC values of 0.755, 0.733, and 0.668 for predicting 1-, 5-, and 10-year overall survival (OS), respectively. (**C)** A nomogram was developed by integrating the MRG score with clinical variables to improve prognostic prediction. (**D**) Calibration curves showed the nomogram's accurate predictions for 3-year and 5-year OS. (**E**) The nomogram exhibited enhanced prognostic performance with AUC values of 0.662, 0.638, and 0.652 for 1-, 3-, and 5-year OS predictions, outperforming the individual MRG score. (**F**) K-M survival analysis confirmed the nomogram's superior prognostic ability (p<0.001).


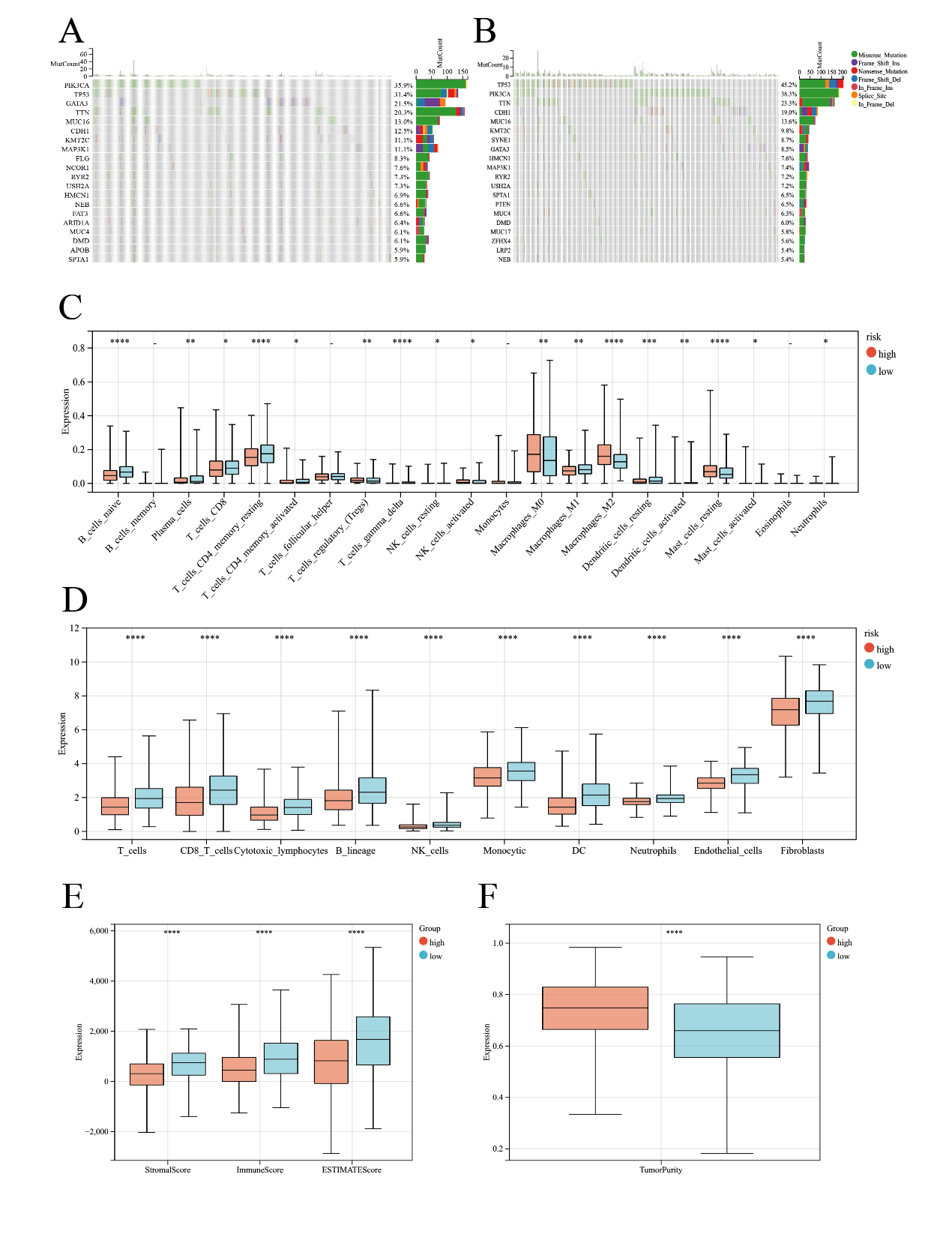


**Supplementary Figure 3. Molecular and immune characteristics of BRCA risk subgroups.​**​ (**A-B**) Mutation landscape: Distinct mutation frequencies of PIK3CA, TP53, GATA3, TTN, and MUC16 between high- and low-risk groups. (**C**) Immune cell infiltration: High-risk group: Enriched Tregs, M0/M2 macrophages, resting mast cells, Low-risk group: Increased naïve B cells, CD8+ T cells, M1 macrophages. (**D**) MCP-counter analysis: Enhanced infiltration of T cells, cytotoxic lymphocytes, B cells, and myeloid cells in low-risk tumors. (**E-F**) ESTIMATE analysis: Low-risk group: Higher stromal/immune scores, lower tumor purity, High-risk group: Opposite pattern observed.


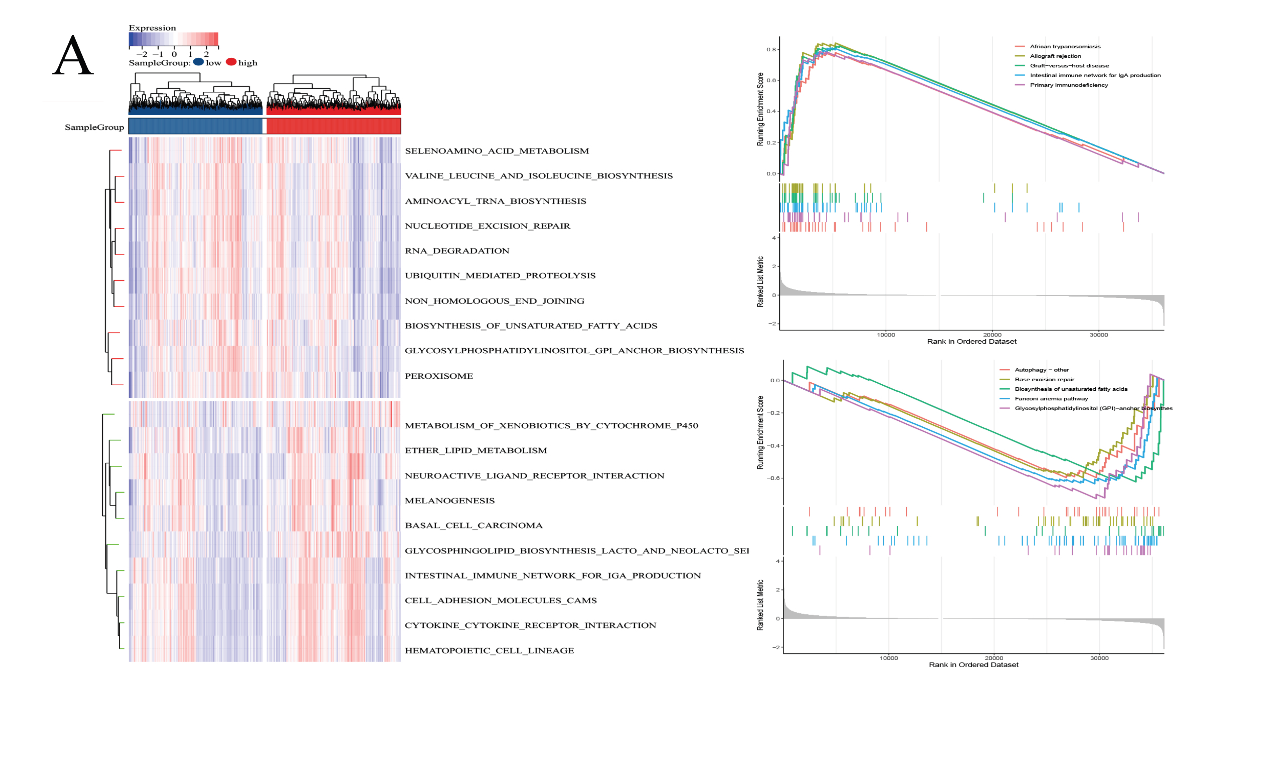


**Supplementary Figure 4. Pathway enrichment analysis of SLPI expression patterns in BRCA.​** (**A**) ​​Activated pathways in SLPI-low tumors:​​ Selenoamino acid metabolism, Aminoacyl tRNA biosynthesis, Ubiquitin-mediated proteolysis. Suppressed pathways in SLPI-low tumors:​​ Xenobiotic metabolism by cytochrome P450, Neuroactive ligand-receptor interactions, Cytokine-cytokine receptor interactions.

| **Oligonucleotides** | **Nucleotide sequence (5'-3')** | |
| --- | --- | --- |
| **Haghl** |  | |
| Si-HAGHL-1  Si-HAGHL-2 | | ACGCTTAGCAACCTGGAGTTT  CAAGCTGTCCTGGGCTAAGAA |
| **Primer** |  | |
| GAPDH | GGCCTCCAAGGAGTAAGACC (forward) | |
|  | AGGGGAGATTCAGTGTGGTG (reverse) | |
| HAGHL | CCCGAGACGAAGGTGTTCTG (forward) | |
|  | CGCAGGAAGGGGTTGTAGAG (reverse) | |
|  |  | |

**Supplementary table 1. Oligonucleotides used in research**
